# Corylin protects LPS-induced sepsis and attenuates LPS-induced inflammatory response

**DOI:** 10.1038/srep46299

**Published:** 2017-04-11

**Authors:** Yung-Li Hung, Shih-Hua Fang, Shu-Chi Wang, Wei-Chung Cheng, Po-Len Liu, Chia-Cheng Su, Chi-Shuo Chen, Ming-Yii Huang, Kuo-Feng Hua, Kun-Hung Shen, Yu-Ting Wang, Katsuhiko Suzuki, Chia-Yang Li

**Affiliations:** 1 Graduate School of Sport Sciences, Waseda University, Tokorozawa 359-1192, Japan; 2 Institute of Athletics, National Taiwan University of Sport, Taichung 40404, Taiwan; 3 Health Management Center, Kaohsiung Medical University Hospital, Kaohsiung Medical University, Kaohsiung 80708, Taiwan; 4 Graduate Institute of Biomedical Sciences, China Medical University, Taichung 40402, Taiwan; 5 Department of Respiratory Therapy, College of Medicine, Kaohsiung Medical University, Kaohsiung 80708, Taiwan; 6 Division of Urology, Department of Surgery, Chi-Mei Medical Center, Tainan 71004, Taiwan; 7 Graduate Institute of Medicine, College of Medicine, Kaohsiung Medical University, Kaohsiung 80708, Taiwan; 8 Department of Biomedical Engineering and Environmental Sciences, National Tsing Hua University, Hsinchu 30013, Taiwan; 9 Department of Radiation Oncology, Cancer Center, Kaohsiung Medical University Hospital, Kaohsiung Medical University, Kaohsiung 80708, Taiwan; 10 Department of Biotechnology and Animal Science, National Ilan University, Ilan 26047, Taiwan; 11 Center for General Education, Southern Taiwan University of Science and Technology, Tainan 71005, Taiwan; 12 Department of Urology, Taipei Medical University, Taipei 11031, Taiwan; 13 Faculty of Sport Sciences, Waseda University, Tokorozawa 359-1192, Japan; 14 Center for Infectious Disease and Cancer Research, Kaohsiung Medical University, Kaohsiung 80708, Taiwan; 15 Department of Medical Research, Kaohsiung Medical University Hospital, Kaohsiung Medical University, Kaohsiung 80756, Taiwan

## Abstract

Corylin is a main compound isolated from *Psoralea corylifolia* L. (Fabaceae). A variety of pharmacological effects such as antioxidant, anti-proliferation, and anti-inflammatory properties of corylin have been reported. Nevertheless, the effect of corylin in microbial infection and sepsis remains unclear. In the present study, we investigated the anti-inflammatory effects of corylin. Our experimental results demonstrated that corylin inhibited the production of TNF-α, IL-6 and NO by both LPS-activated RAW 264.7 cells and LPS-activated murine peritoneal macrophages. Moreover, corylin suppressed the expression levels of iNOS and COX-2, reduced the production of PGE_2_ and HMGB1, blocked the translocation of HMGB1 from the nucleus to cytosol, and decreased the phosphorylation of MAPKs in LPS-activated RAW 264.7 cells as well as suppressed the activity of NF-κB in LPS-activated J-Blue cells. In addition, the administration of corylin reduced the production of NO and TNF-α, decreased LPS-induced liver damage markers (AST and ALT) and kidney damage markers (BUN and CRE), attenuated infiltration of inflammatory cells and tissue damage of lung, liver and kidney, and enhanced the survival rate of LPS-challenged mice. Taken together, these results show the anti-inflammatory properties of corylin on LPS-induced inflammation and sepsis. Corylin could potentially be a novel anti-inflammatory and immunosuppressive drug candidate in the treatment of sepsis and septic shock.

The natural products from traditional herbal medicine have potential for investigation of new anti-inflammatory drugs^1^,^2^,^3^,^4^. The *Psoralea corylifolia* L. (Fabaceae) has been used in Ayurvedic medicine and traditional Chinese medicine^5^, and has been recommended in the treatment of several diseases such as skin diseases, cardiovascular diseases and osteoporosis^6^. The extracts of *Psoralea corylifolia* L. have been shown to possess anti-bacterial, anti-oxidative, anti-diabetic, anti-tumor and immunomodulatory effects^7^,^8^,^9^,^10^. Corylin is a main compound isolated from the whole plant, fruit and seed of *Psoralea corylifolia* L.^5^, and exhibits pharmacological effects in regulating antioxidant activity^11^ and osteoblastic proliferation-stimulating activity^12^. Furthermore, corylin inhibits interleukin-6 (IL-6)-induced signal transducer and activator of transcription 3 (STAT3) activity in hepatocarcinoma Hep3B cells^13^. However, the anti-inflammatory effects of corylin on LPS-stimulated macrophages and LPS-induced sepsis in mice remains unclear.

Inflammation is not only associated with the innate immune response to infection, but is also involved in the pathogenesis of several diseases such as metabolic syndrome, type 2 diabetes, atherosclerosis and cancer^14^,^15^,^16^. Macrophage, a main type of antigen presenting cell, is widely distributed in the body and plays a critical role in modulating inflammatory response and regulate the pathogenesis of these diseases^17^,^18^. Several pro-inflammatory cytokines such as tumor necrosis factor-α (TNF-α), IL-1β and IL-6, and pro-inflammatory mediators, nitric oxide (NO) and prostaglandins (PGs), are secreted from the activated macrophages. NO is synthesized from L-arginine by inducible NO synthase (iNOS), and exerts anti-microbial and inflammatory effects^19^, but overproduction of NO causes damage to various tissues^20^,^21^. In addition, PGs are metabolized from arachidonic acid through cyclooxygenase (COX)-2, and converted to prostaglandin E_2_ (PGE_2_) to mediate inflammatory response^22^. Although the inflammatory response is a defense mechanism against infection, systemic inflammatory response leads to multiple organ failure or death, such as sepsis and septic shock^23^. Additionally, it has been demonstrated that TNF-α and IL-1β are early pro-inflammatory mediators and high mobility group box 1 (HMGB1) is a late pro-inflammatory mediator in the pathogenesis of sepsis^24^. HMGB1 is a DNA-binding nuclear protein that translocates to cytosol and releases to extracellular fluid by activated macrophages^25^. Extracellular HMGB1 is produced as a damage-associated molecular pattern molecule (DAMP) recognized by Toll-like receptor (TLR)-4 and TLR-2 in innate immune cells, and induces the production of pro-inflammatory mediators in macrophages^26^.

TLR-4 is the receptor for lipopolysaccharide (LPS), a major component of the outer membrane of Gram-negative bacteria. Activation of the TLR-4 signaling-pathway plays an important role in regulating the secretion of pro-inflammatory cytokines and mediators through its downstream signaling pathway including mitogen-activated protein kinase (MAPK) and nuclear factor-κB (NF-κB) pathways in macrophages^27^. The MAPK pathways include JUN N-terminal kinase (JNK) 1/2, p38 MAPK and extracellular-signal-regulated kinases (ERK) 1/2 pathways, which play important roles in regulating activation of NF-κB and activator protein-1 (AP-1)^28^, and synthesis of pro-inflammatory cytokine and mediator production in response to stimulation of LPS^29^,^30^. Hence inhibition of MAPK pathways leads to attenuate the production of pro-inflammatory cytokines and mediators.

In the present study, we investigated the anti-inflammatory effects of corylin on LPS-stimulated RAW 264.7 cells and mouse peritoneal macrophages. In addition, we used an experimental LPS-induced sepsis model for studying the anti-inflammatory effects of corylin *in vivo*. Our results demonstrated that corylin suppresses LPS-induced production of pro-inflammatory cytokines and mediators in RAW 264.7 cells and mouse peritoneal macrophages. Corylin exerts anti-inflammatory effect via downregulation of MAPK pathways and NF-κB activity. Furthermore, administration of corylin protected mice from endotoxin-induced tissue injury and death, and blocked the production of NO and TNF-α in serum of LPS-challenged mice.

## Results

### Corylin reduces the production of PGE_2_ and NO and suppresses the expression of COX-2 and iNOS in LPS-activated RAW 264.7 cells

**Figure 1:**
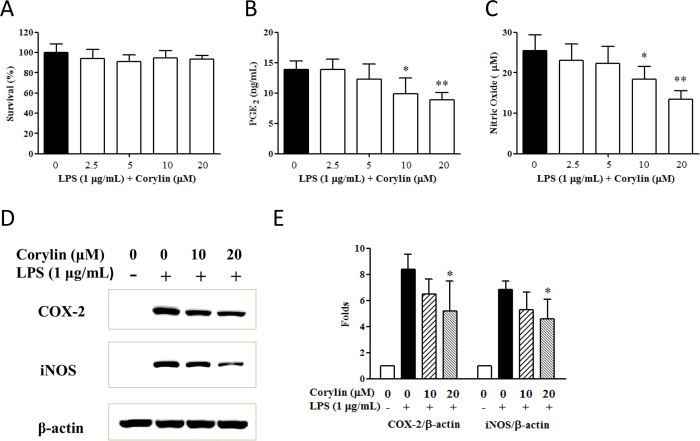
Effects of corylin on the cell viability and the production of PGE_2_ and NO by LPS-activated RAW 264.7 cells. Cells were incubated with different concentrations of corylin for 1 h, and then were treated with LPS (1 μg/mL) for 24 h. (**A**) The cell viability was determined by the MTT assay. (**B**) The level of PGE_2_ in the cell culture supernatant was measured by PGE_2_ ELISA (**C**) The level of NO in the cell culture supernatant was measured by the Griess reaction. (**D**) Cells were incubated with different concentrations of corylin for 1 h, and then were treated with LPS (1 μg/mL) for 6 h. The expressions of COX-2 and iNOS were determined by western blot. (**E**) Band intensities were quantified from three independent experiments. The expression ratio represents the change in the ratio relative to β-actin compared with the untreated control group. The data are presented as the means ± SD of three independent experiments. Statistical significance was assessed by one-way ANOVA followed by Tukey post-hoc test and represented as follows: *p < 0.05 and **p < 0.01 vs. LPS alone.

To avoid the toxic effects of corylin, we firstly investigated the effect of corylin on the cell survival of RAW 264.7 cells. Cells were pre-treated with corylin at various concentrations (0 μM to 20 μM) for 1 h, and then treated with LPS 1 μg/mL for 24 h. Cell viability was analyzed by the MTT assay. The results revealed that the cell survival rate did not differ significantly when the RAW 264.7 cells were treated with corylin 0~20 μM (Fig. 1A). To investigate the effect of corylin on LPS-induced PGE_2_ and NO production, RAW 264.7 cells were pre-treated with corylin in non-cytotoxicity dosages (0~20 μM) for 1 h, and then stimulated with LPS (1 μg/mL) for 24 h. As shown in Fig. 1B and C, corylin significantly inhibited the production of PGE_2_ and NO by LPS-stimulated RAW 264.7 cells in a concentration-dependent manner. In addition, our results showed that corylin decreased the expression of COX-2 and iNOS by western blot, as compared with LPS alone (Fig. 1D and E).

### Corylin inhibits LPS-induced production of pro-inflammatory cytokines in RAW 264.7 cells

**Figure 2:**
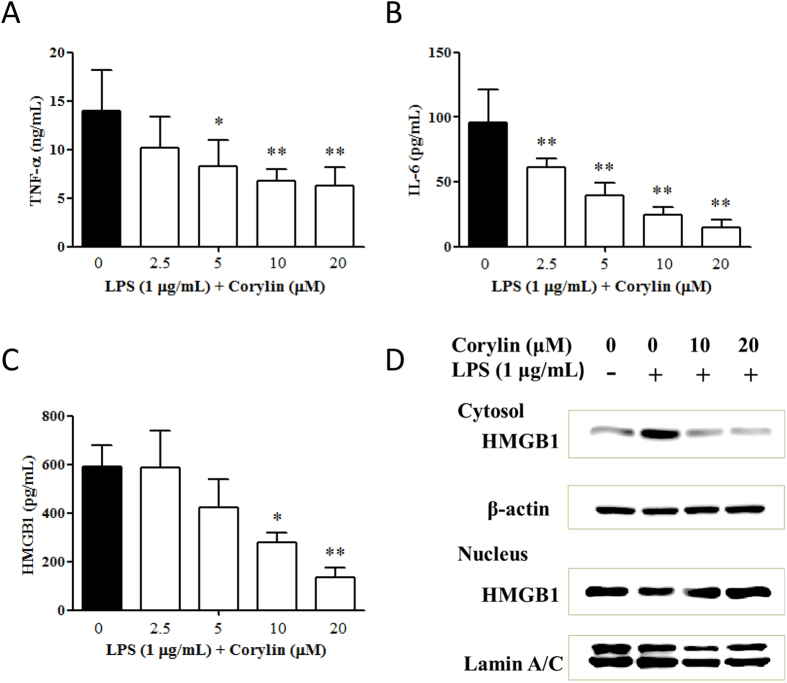
Effects of corylin on the production of TNF-α, IL-6 and HMGB1 by LPS-activated RAW 264.7 cells. Cells were incubated with different concentrations of corylin for 1 h, and then were treated with LPS (1 μg/mL) for 24 h. The levels of (**A**) TNF-α and (**B**) IL-6 (**C**) HMGB1 in the cell culture supernatant were measured by ELISA. The data are presented as the means ± SD of three independent experiments. Statistical significance was assessed by one-way ANOVA followed by Tukey post-hoc test and represented as follows: *p < 0.05 and **p < 0.01 vs. LPS alone. (**D**) Cells were incubated with different concentrations of corylin for 1 h, and then were treated with LPS (1 μg/mL) for 6 h. The expression of HMGB1 in both cytosol and nucleus was determined by western blot. The Western blotting results are representative of results obtained in three separate experiments.

It has been well recognized that TNF-α and IL-6 are critical pro-inflammatory cytokines in response to LPS^31^. On the other hand, DNA-binding nuclear protein HMGB1 is also recognized as a crucial pro-inflammatory cytokine in several inflammatory diseases^26^. RAW 264.7 cells were pre-treated with various concentrations of corylin for 1 h, and then treated with LPS (1 μg/mL) for 24 h. The experimental results showed that corylin decreased the production of TNF-α, IL-6 and HMGB1 by LPS-activated RAW 264.7 cells in a concentration-dependent manner. (Fig. 2A–C). Moreover, our results indicated that corylin inhibited the translocation of HMGB1 from the nucleus to cytosol by western blot, as compared with LPS alone (Fig. 2D).

### Corylin decreases the production of NO and pro-inflammatory cytokines in LPS-activated murine peritoneal macrophages

**Figure 3:**
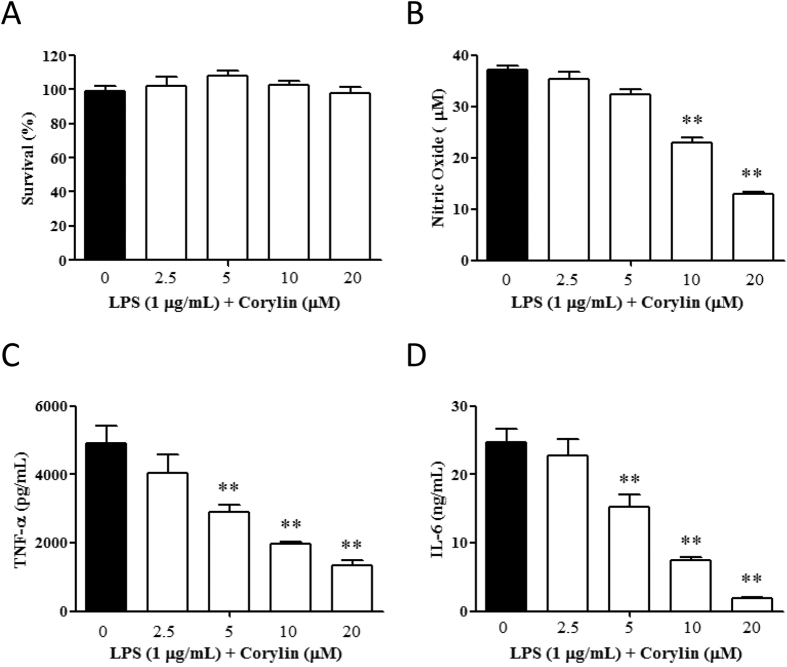
Effects of corylin on the cell viability and the production of NO, TNF-α and IL-6 by LPS-activated murine peritoneal macrophages. Thioglycollate-elicited macrophages were incubated with different concentrations of corylin for 1 h and then treated with 1 μg/mL LPS for 24 h. (**A**) The cell viability was determined by the MTT assay. (**B**) The level of NO in the cell culture supernatant was measured by the Griess reaction. (**C,D**) The levels of TNF-α and IL-6 in the cell culture supernatant were measured by ELISA. The data are presented as the means ± SD. Statistical significance was assessed by one-way ANOVA followed by Tukey post-hoc test and represented as follows: *p < 0.05 and **p < 0.01 vs. LPS alone.

The above experiments demonstrated that corylin exhibited anti-inflammatory activities through inhibiting the production of NO, TNF-α, and IL-6 by LPS-activated RAW 264.7 cells. We further used murine peritoneal macrophages to verify the anti-inflammatory activities of corylin. Thioglycollate-elicited macrophages were incubated with different concentrations of corylin (0~20 μM) for 1 h, and then treated with LPS (1 μg/mL) for 24 h. As shown in Fig. 3A, cell survival rate did not differ significantly when the murine peritoneal macrophages were treated with 0~20 μΜ corylin. In addition, our experimental results indicated that corylin significantly inhibits the production of NO, TNF-α and IL-6 by LPS-activated murine peritoneal macrophages (Fig. 3B–D).

### Corylin decreases the phosphorylation of MAPKs by LPS-activated RAW 264.7 cells and suppresses activation of NF-κB by LPS-activated J-Blue cells

**Figure 4:**
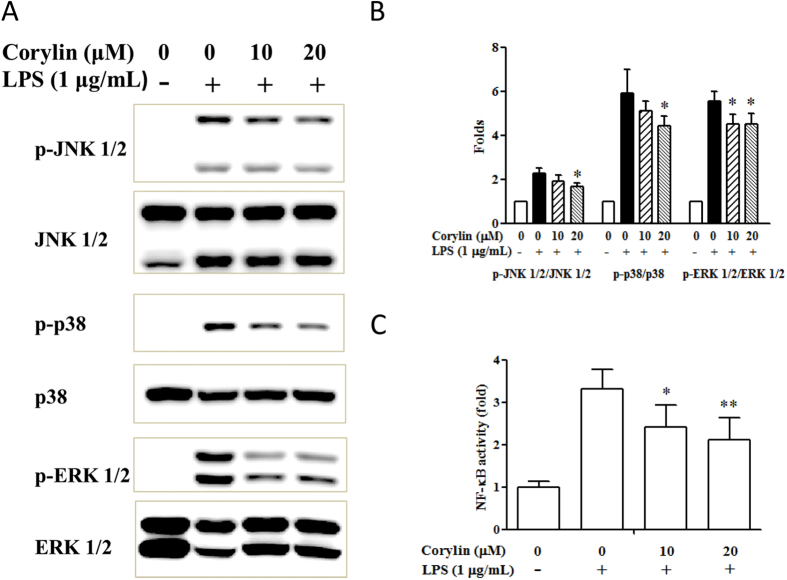
Effects of corylin on the expression and phosphorylation of JNK 1/2, p38 MAPK and ERK 1/2 by LPS-activated RAW 264.7 cells and the activation of NF-κB by LPS-activated J-Blue cells. Cells were incubated with different concentrations of corylin for 1 h, and then were treated with LPS (1 μg/mL) for 30 min. (**A**) The expression levels of phosphor-JNK 1/2, JNK 1/2, phosphor-p38 MAPK, p38 MAPK, phosphor-ERK 1/2 and ERK 1/2 were determined by western blot. (**B**) Band intensities were quantified from three independent experiments. The relative fold of phosphorylation activity was normalized to that of the un-phosphorylated form and compared to untreated samples. (**C**) J-Blue cells were incubated with different concentrations of corylin for 1 h, and then were treated with LPS (1 μg/mL) for 24 h. The activity of NF-κB was measured by NF-κB promoter reporter assay. The data are presented as the means ± SD of three independent experiments. Statistical significance was assessed by one-way ANOVA followed by Tukey post-hoc test and represented as follows: *p < 0.05 and **p < 0.01 vs. LPS alone.

MAPKs (JNK 1/2, p38 MAPK and ERK 1/2) are activated by inflammation, and have been implicated as key factors in regulating the production of pro-inflammatory mediators and cytokines^28^,^29^. Thus, we examined whether corylin regulates the expression of MAPKs by LPS-activated RAW 264.7 cells. As shown in Fig. 4A and B, the phosphorylation of JNK 1/2, p38 MAPK and ERK 1/2 were markedly increased after LPS stimulation. Treatment of corylin (10 and 20 μM) significantly suppressed LPS-induced phosphorylation of JNK 1/2, p38 MAPK and ERK 1/2 in RAW 264.7 cells (Fig. 4A and B). Furthermore, corylin significantly inhibited the activation of NF-κB by LPS-activated J-Blue cells (Fig. 4C).

### Corylin exhibits protective effects against LPS-induced tissue damage in LPS-challenged mice

**Figure 5:**
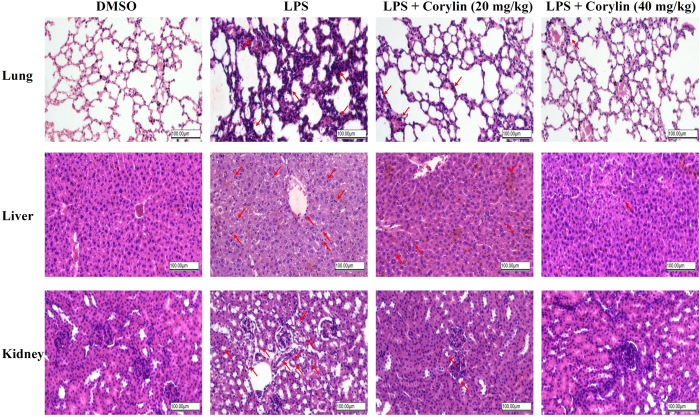
Effects of corylin on the tissue damage of lung, liver and kidney in LPS-challenged mice. Female C57BL/6 mice were injected with corylin (20 or 40 mg/kg, i.p.) or vehicle (DMSO) 1 h before LPS injection (50 mg/kg, i.p.), tissues of lung, liver and kidney were harvested 20 h after LPS injection. The results show H&E-staining of lung, liver or kidney tissue sections from the indicated group (×200). The damage sites and infiltration of inflammatory cells are indicated by red arrows. The figure is a representative of three independent experiments.

The systemic inflammatory response syndrome (SIRS) and sepsis might be caused by infection of bacteria. Endotoxin, LPS is a critical factor to induce severe and systemic inflammatory response and acute tissue injury on such organs as the lung, liver and kidney during sepsis^32^. We further investigated the anti-inflammatory effects of corylin using LPS-challenged mice. Mice were pre-treated with corylin (20 or 40 mg/kg, i.p.) for 1 h, and then injected with LPS (50 mg/kg, i.p.) to induce experimental sepsis. The lung, liver and kidney samples were collected and examined by histopathology; the blood samples were collected and the levels of AST, ALT, BUN and CRE were measured. LPS administration markedly increased inflammatory cells infiltration in lung, liver and kidney tissue samples (Fig. 5). In LPS-induced lung injury, the thickness of alveolar wall was increased, and the number of pulmonary alveolus was reduced by LPS injection. The administration of corylin repressed the swelling of alveolar wall and declined the number of pulmonary alveolus in LPS-challenged mice. For liver injury by LPS, the administration of corylin suppressed LPS-induced infiltration of inflammatory cells into the cavities of liver tissue. For the kidney injury, renal tubular epithelial cells were sloughed, and brush borders and renal epithelial cells were decreased after LPS injection. The administration of corylin inhibited the sloughing of tubular epithelial cells and diminished brush borders and epithelial cells in the kidney. In addition, liver damage markers (AST and ALT) and kidney damage markers (BUN and CRE) were markedly increased in LPS-challenged mice. Administration of 20 mg/kg and 40 mg/kg corylin significantly reduced AST, ALT, BUN and CRE in LPS-challenged mice (Table 1). Taken together, corylin exhibited protective effects against endotoxin-induced tissue damage *in vivo*.

### Corylin enhances the survival rate of LPS-challenged mice and inhibits the production of NO and TNF-α in mouse serum

**Figure 6:**
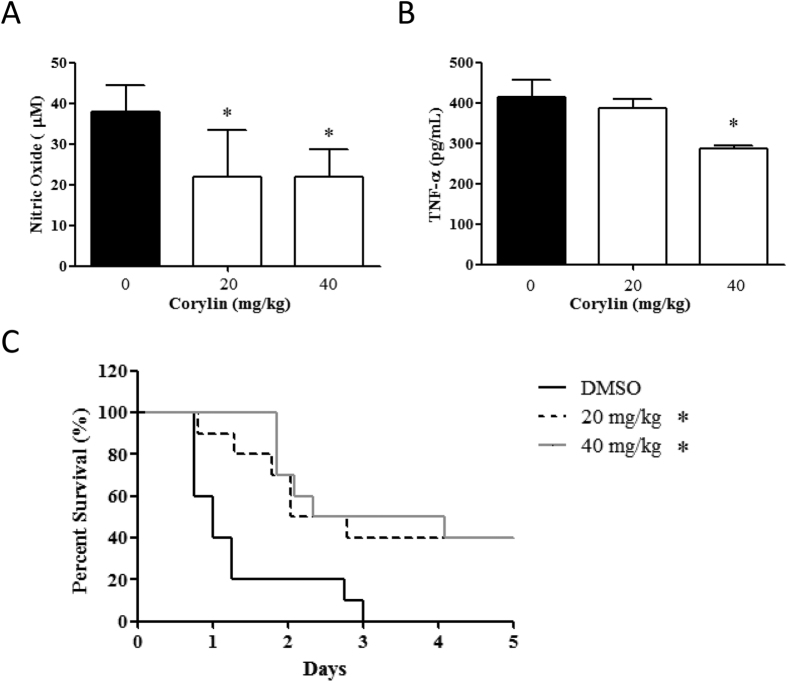
Effects of corylin on mortality and serum NO and TNF-α levels in LPS-challenged mice. Female C57BL/6 mice were injected with corylin (20 or 40 mg/kg, i.p.) or vehicle (DMSO) 1 h before LPS injection (50 mg/kg, i.p.), and the blood samples were harvested 4 h after LPS injection. (**A**) The level of NO in the mouse serum was measured by the Griess reaction. (**B**) The level of TNF-α in the mouse serum was measured by ELISA. (**C**) The survival was recorded at different intervals. Each group contained 10 mice. Statistical significance was assessed by Log-Rank test and represented as follows: *p < 0.05 vs. DMSO.

The overwhelming production of pro-inflammatory cytokines and mediators results in tissue damage or lethality. Since the above experimental results indicated corylin exhibited protective effects against LPS-induced tissue damage in LPS-challenged mice, we further examined whether corylin affected LPS-induced septic lethal rate and production of NO and TNF-α in LPS-challenged mice. Mice were pre-treated with corylin (20 or 40 mg/kg, i.p.) for 1 h, and then injected with LPS (50 mg/kg, i.p.). After LPS injection for 4 h, we collected the blood samples and analyzed the levels of NO and TNF-α. The survival was recorded for 5 days. The results showed that administration of corylin significantly decreased the production of NO and TNF-α in serum after LPS challenge (Fig. 6A and B). Moreover, corylin also increased survival rate compared to LPS alone (Fig. 6C).

## Discussion

Although *Psoralea corylifolia* L. has been reported to exert several biological activities such as anti-oxidative, anti-diabetic, anti-tumor and immunomodulatory effects^8^,^9^,^10^, the anti-inflammatory effect remains unclear. Corylin is a main compound that is isolated from *Psoralea corylifolia* L., has potent antioxidant activity^11^ and osteoblastic proliferation-stimulating activity^12^. Notably, corylin exhibits potent anti-inflammatory activity on IL-6-stimulated hepatocarcinoma Hep3B cells through suppressed IL-6-induced phosphorylation of STAT3^13^. In this study, we firstly demonstrated that corylin exhibited inhibitory effects on LPS-induced inflammation and had potential in the prevention and treatment for LPS-induced sepsis.

NO is markedly produced in inflammatory response of LPS-stimulated macrophages, and then peroxynitrite (ONOO^-^) is synthesized. A large amount of cytotoxic ONOO^-^ leads to tissue damage by oxidative stress and DNA damage^33^. In addition, COX-2 is a key factor for synthesizing PGs, and mediates inflammatory response resulting in fever, pain, hypersensitivity, and edema^22^. Many natural products that exhibit inhibitory effect on NO and PGE_2_ production and suppress iNOS and COX-2 have been found^34^. Our data revealed that corylin dramatically decreased LPS-induced NO and PGE_2_ production in RAW 264.7 cells, mouse peritoneal macrophages and LPS-challenged mice, as well as inhibited the expression of iNOS and COX-2 in LPS-activated RAW 264.7 cells.

Macrophages play important roles in host defense to infection, repair of damaged tissue, and secretion of pro-inflammatory cytokines such as TNF-α and IL-6 to modulate inflammatory response^35^. However, overwhelming secretion of pro-inflammatory cytokines causes severe tissue damage, multiple organ failure or death^23^. Therefore, repressing overproduction of pro-inflammatory cytokines is a therapeutic strategy for controlling inflammatory diseases. Furthermore, it had been indicated that HMGB1 is a crucial late pro-inflammatory mediator in septic lethality. Decreasing HMGB1 level has been considered as a wider therapeutic window for sepsis, and HMGB1 inhibitors have been demonstrated to protect mice from sepsis^36^,^37^. Our results indicated that corylin inhibited the production of TNF-α and IL-6 in both LPS-activated RAW 264.7 cells and LPS-activated murine peritoneal macrophages in a concentration-dependent manner. Additionally, our results also showed that corylin suppressed the secretion of HMGB1 and blocked HMGB1 translocation from the nucleus to cytosol in LPS-activated RAW264.7 cells.

LPS-triggered activation of MAPKs cascade is a critical signal transduction in modulating the production of inflammatory cytokines and mediators, and regulates activation of NF-κB and AP-1^28^,^29^. In the present study, we demonstrated that corylin inhibited the phosphorylation of JNK 1/2, p38 MAPK and ERK 1/2 in LPS-activated RAW 264.7 cells and decreased the NF-κB activity in LPS-activated J-Blue cells. In consistency with the production of inflammatory cytokines (TNF-α, IL-6 and HMGB1) and mediators (NO and PGE_2_), we suggest corylin suppressed LPS-induced pro-inflammatory cytokines and mediators via inhibition of MAPKs and NF-κB signaling pathways.

LPS and other microbial compounds are involved in the pathogenesis of sepsis and stimulate immune responses, leading to tissue damage and multiple organ failure^38^. Management of overwhelming inflammatory response is an important issue for the treatment of sepsis^39^. In the present study, we performed a series of examinations to test the anti-inflammatory activities of corylin using LPS-challenged mice. Our results showed that administration of corylin inhibits infiltration of inflammatory cells in lung, liver and kidney and decreased the loss of pulmonary cells, hepatic cells and renal cells in LPS-challenged mice. In addition, administration of corylin reduced the levels of AST, ALT, BUN, and CRE in LPS-challenged mice. These results indicated that corylin has protective effects against LPS-induced acute liver and kidney injury. Furthermore, corylin suppressed NO and TNF-α production in LPS-challenged mice. Collectively, these findings suggest that corylin abates LPS-induced tissue damage via blocked NO and TNF-α production in the acute phase of LPS challenge.

The mortality rate of sepsis was approximately 30%, and it was up to 50% for severe sepsis and 80% for septic shock^40^. To reduce the mortality rate of sepsis is an important issue. In our experimental septic animal model, LPS challenge (50 mg/kg) resulted in 100% mortality rate with 3 days. Administration of corylin not only extended the survival time but also increased the survival rate to 40%.

In summary, this is the first report illustrating that corylin has anti-inflammatory effects in LPS-induced inflammation and has protective effects in LPS-induced septic shock. We used two murine macrophages (*in vitro*, RAW 264.7 macrophages; *ex vivo*, murine peritoneal macrophages) and a septic animal model to study the anti-inflammatory effects of corylin. Our results demonstrated that corylin inhibited the expression of LPS-induced iNOS and COX-2, decreased the production of LPS-induced TNF-α, IL-6, HMGB1, NO and PGE_2_, suppressed the phosphorylation of MAPKs, decreased the activation of NF-κB, protected LPS-induced tissue damage, and increased the survival rate from LPS-induced septic shock. These results provide new insights into the mechanism of anti-inflammatory effects of corylin, and a new indication for corylin for the treatment of sepsis.

## Methods

### Ethics Statement

This study was undertaken according to the recommendations in the Guide for the Care and Use of Laboratory Animals of the National Institutes of Health and approved by the Committee on the Ethics of Animal Experiments of the Kaohsiung Medical University (Permit Number: 104092).

### Reagents

RPMI-1640 medium, penicillin, streptomycin and fetal bovine serum (FBS) were purchased from Gibco-BRL (Life Technologies, Grand Island, NY, USA). LPS (from Escherichia coli 0111:B4), Griess reagent, bovine serum albumin (BSA), phosphate-buffered saline (PBS), RIPA buffer, protease inhibitor cocktail, phosphatase inhibitor cocktail, stripping buffer, thioglycollate medium, trypan blue, 3-(4,5-dimethylthiazol-2-yl)-2, 5-diphenyl tetrazolium bromide (MTT), hematoxylin solution, and eosin solution were purchased from Sigma Aldrich (St. Louis, MO, USA). Limulus amebocyte lysate (LAL) single test vial with a sensitivity limit of 0.03 EU/mL was purchased from Associates of Cape Cod, Inc. (Falmouth, MA, USA). Both TNF-α and IL-6 enzyme-linked immunosorbent assay (ELISA) kits were purchased from eBioscience (San Diego, CA, USA). PGE_2_ ELISA kit was obtained from Cayman Chemical Company (Ann Arbor, MI, USA). HMGB1 ELISA kit was purchased from Aviva Systems Biology (San Diego, CA, USA). BCA protein assay reagent was purchased from Thermo Scientific (Waltham, MA, USA). Nuclear protein isolation-translocation assay kit was purchased from Fivephoton Biochemicals (San Diego, CA, USA). For western blotting, rabbit antibodies against mouse phospho-JNK1/2, JNK1/2, phospho-p38 MAPK, and p38 MAPK, and phospho-ERK1/2, ERK1/2 were purchased from Cell Signaling (Farmingdale, NY, USA). Rabbit antibodies against mouse iNOS, mouse antibodies against mouse COX-2 and secondary antibodies were obtained from Santa Cruz Biotechnology (Santa Cruz, CA, USA). Mouse antibodies against mouse HMBG1 was purchased from OriGene Technologies, Inc. (Rockville, MD, USA). Rabbit antibodies against mouse Lamin A/C was purchased from GeneTex, Inc. (Irvine, CA, USA). Corylin was purchased from ChemFaces (Wuhan, Hubei, China). To avoid potential of endotoxin contamination, corylin was measured by the LAL assay. Results indicated that endotoxin level in corylin obtained less than 0.03 EU/mL.

### Cells and cell culture

The murine macrophage cell line (RAW 264.7) was purchased from Bioresource Collection and Research Center (Food Industry Research and Development Institute, Hsinchu, Taiwan), and was cultured in RPMI-1640 medium supplemented with antibiotics (100 U/mL penicillin and 100 U/mL streptomycin) and 10% (v/v) FBS in a humidified atmosphere of 5% CO_2_ at 37 °C and passaged every 2–3 days to maintain growth.

### Isolation of peritoneal macrophages

Thioglycollate-elicited peritoneal macrophages were collected from specific pathogen-free female C57BL/6 mice at 6~8 week of age by intraperitoneal injection (i.p) of 1 mL sterile 3% thioglycollate medium for 4 day prelavage with 10 mL PBS. Cells were washed once with PBS and resuspended in RPMI-1640 medium at a density of 1 × 10^6^ cells/mL. The peritoneal macrophages had a mean viability of 95% as judged by the trypan blue dye exclusion assay. To allow macrophage adherence, cells were plated and incubated in a 5% CO_2_ incubator for 3 h at 37 °C.

### Measurement of NO

The concentration of NO in culture supernatant was determined as nitrite by the Griess reagent. RAW 264.7 cells were seeded in a 96-well plate at a concentration of 1 × 10^5^ cells/mL, and were allowed to acclimatize overnight. The cells were pre-treated with various concentrations of corylin (0 μM to 20 μM) for 1 h, and then were treated with LPS (1 μg/mL) for 24 h. The supernatant of cell culture was collected and assayed the concentration of NO using the Griess reagent according to the manufacturer’s protocol (Sigma Aldrich, St. Louis, MO, USA). The concentration of nitrite was converted into sodium nitrite concentration as a standard.

### MTT assay for cell viability

Cells were seeded in a 96-well plate at a concentration of 1 × 10^5^ cells/mL, and were allowed to acclimatize overnight. The cells were pre-treated with various concentrations of the corylin (0 μM to 20 μM) for 1 h, and then treated with LPS 1 μg/mL for 24 h. Cell viability was measured by the ability of viable cells to reduce MTT to formazan based on the ability of living cells to utilize Thiazole Blue and convert it into purple formazan, which absorbs light at 570 nm and could be determined by spectrophotometrically. The results are normalized to the untreated control.

### Cytokine measurement

The production of TNF-α, IL-6, PGE_2_ and HMGB1 were measured by ELISA. RAW 264.7 cells were seeded in 96-well plate at a concentration of 1 × 10^5^ cells/mL, and were allowed to acclimatize overnight. The cells were pre-treated with various concentrations (0 μM to 20 μM) of corylin for 1 h, and then treated with LPS 1 μg/mL for 24 h. The supernatant of cell culture was used for determination of cytokine concentration by ELISA according to the manufacturer’s protocol.

### Western blotting

Cells were lysed by RIPA buffer with protease inhibitors and phosphatase inhibitors according to the manufacturer’s protocol (Sigma Aldrich, St. Louis, MO, USA). For the isolation of nucleus and cytosol proteins, the nuclear protein isolation-translocation assay kit was employed according to the manufacturer’s protocol. The protein concentration was determined using the BCA protein assay reagent according to the manufacturer’s instructions (Thermo Scientific, Waltham, MA, USA). Cellular protein extracts were separated by electrophoresis using 8% SDS polyacrylamide gel and were electro-blotted onto PVDF membranes. The membranes were incubated with blocking solution for 1 h at room temperature, followed by incubation overnight with primary antibodies at 4 °C (1:1000). Blots were washed three times with Tris-buffered saline/Tween 20 (TBST) and incubated with a 1:5000 dilution of horseradish peroxidase conjugated secondary antibody for 1 h at room temperature. Blots were again washed three times with TBST and developed using an ECL chemiluminescence substrate (Thermo Scientific, Waltham, MA, USA). Band intensities were quantified using Alphaview SA software (Alpha Innotech Corporation, San Leandro, CA, USA).

### NF-κB promoter reporter assay

J-Blue cell, a J774A.1 macrophage cell line, is stably expressing the gene for secreted embryonic alkaline phosphatase (SEAP) inducible by NF-κB^41^, which was maintained in RPMI 1640 medium supplemented with Zeocin (200 μg/mL) (InvivoGen, San Diego, CA, USA). Cells were seeded in a 96-well plate at a density of 1 × 10^6^ cells/mL, and grown overnight in a 5% CO_2_ incubator at 37 °C. The cells were pre-treated with various concentrations of corylin (10 μM and 20 μM) for 1 h, and then were treated with LPS (1 μg/mL) for 24 h. The medium then was harvested and mixed with QUANTI-Blue medium (20 μL cell culture supernatant to 200 μL QUANTI-Blue medium) (InvivoGen, San Diego, CA, USA) in 96-well plates and incubated at 37 °C for 45 min. SEAP activity then was assessed by measuring the optical density at 655 nm using an ELISA reader.

### Animals and experimental sepsis

Female C57BL/6 mice were purchased from the BioLASCO Taiwan., Co. Ltd (Taipei, Taiwan). The animals received LPS in sterile PBS by intraperitoneal injection. In the treatment group, mice were intraperitoneally injected with corylin 20 mg/kg body weight or 40 mg/kg body weight for 1 h before LPS injection (50 mg/ kg body weight). Mice in the control group were administrated with equivalent volume of vehicle (DMSO) for 1 h before LPS injection. Blood samples were collected for 4 h after LPS injection for the determination of NO. The survival states of different groups were recorded at different intervals.

### Histopathology and biochemistry analysis

Female C57BL/6 mice were administrated corylin (20 mg/kg body weight or 40 mg/kg body weight) or equivalent volume of vehicle (DMSO) for 1 h as indicated, and then intraperitoneally injected with LPS (50 mg/kg body weight). After 20 h, mice were sacrificed. Tissues (lung, liver and kidney) were collected and fixed with 4% formaldehyde, and then embedded by paraffin. The tissues were sliced and stained with hematoxylin and eosin (H&E). Blood samples were collected and detected the levels of Alanine Aminotransferase (ALT), Aspartate Aminotransferase (AST), blood urea nitrogen (BUN) and creatinine (CRE) using a Fuji Dri-Chem 3500i Biochemistry Analyzer (Fujifilm Ltd, Japan).

### Statistical analysis

All results are expressed as means ± standard deviation (SD). Each value is the mean of three independent experiments. Statistical analysis was performed using GraphPad Prism 5 (San Diego, CA, USA). For *in vitro* and *in vivo* data, statistical analysis was performed using a one-way ANOVA followed by Tukey post-hoc test, and survival rates between multiple groups were analyzed using Log-Rank test. The significant difference was set at *p < 0.05; **p < 0.01.

## Additional Information

**How to cite this article**: Hung, Y.-L. *et al*. Corylin protects LPS-induced sepsis and attenuates LPS-induced inflammatory response. *Sci. Rep.* **7**, 46299; doi: 10.1038/srep46299 (2017).

**Publisher's note:** Springer Nature remains neutral with regard to jurisdictional claims in published maps and institutional affiliations.

## Figures and Tables

**Table 1:** The effect of corylin on LPS-induced liver and kidney damage markers.

	Control	DMSO	Corylin 20 mg/kg	Corylin 40 mg/kg
AST	200 ± 3	619.75 ± 97.39^^**^^	333.5 ± 31.25^^+^^	289 ± 34.53^^+^^
ALT	46.5 ± 0.5	291.33 ± 85.61^^*^^	72.4 ± 5.61^^+^^	69.33 ± 4.18^^+^^
BUN	28.4 ± 1.2	150.5 ± 11.93^^**^^	41.66 ± 5.44^^++^^	40.3 ± 9.90^^++^^
CRE	0.31 ± 0.01	0.93 ± 0.21^^**^^	0.26 ± 0.04^^++^^	0.28 ± 0.02^^++^^

Female C57BL/6 mice were injected with corylin (20 or 40 mg/kg, i.p.) or vehicle (DMSO) 1 h before LPS injection (50 mg/kg, i.p.), and the blood samples were harvested 20 h after LPS injection. The levels of ALT, AST, BUN and CRE were measured (n = 5). Statistical significance was assessed by one-way ANOVA followed by Tukey post-hoc test and represented as follows: *p < 0.05, **p < 0.01 vs. Control; ^+^*p* < 0.05, ^++^*p* < 0.05 vs. DMSO.
